# Spatial Relationships between Polychaete Assemblages and Environmental Variables over Broad Geographical Scales

**DOI:** 10.1371/journal.pone.0012946

**Published:** 2010-09-23

**Authors:** Lisandro Benedetti-Cecchi, Katrin Iken, Brenda Konar, Juan Cruz-Motta, Ann Knowlton, Gerhard Pohle, Alberto Castelli, Laura Tamburello, Angela Mead, Tom Trott, Patricia Miloslavich, Melisa Wong, Yoshihisa Shirayama, Claudio Lardicci, Gabriela Palomo, Elena Maggi

**Affiliations:** 1 Department of Biology, University of Pisa, CoNISMa (National Interuniversity Consortium of Marine Sciences), Pisa, Italy; 2 School of Fisheries and Ocean Sciences, University of Alaska Fairbanks, Fairbanks, Alaska, United States of America; 3 Departamento de Estudios Ambientales, Centro de Biodiversidad Marina, Universidad Simon Bolivar, Caracas, Venezuela; 4 Atlantic Reference Centre, Huntsman Marine Science Centre, St. Andrews, Canada; 5 Department of Zoology, University of Cape Town, Cape Town, South Africa; 6 Department of Biology, Suffolk University, Boston, Massachusetts, United States of America; 7 Bedford Institute of Oceanography, Dartmouth, Nova Scotia, Canada; 8 Seto Marine Biological Laboratory, Kyoto University, Shirahama, Japan; 9 Laboratorio de Ecosistemas Costeros, Museo Argentino de Ciencias Naturales “Bernardino Rivadavia”, Buenos Aires, Argentina; National Institute of Water & Atmospheric Research (NIWA), New Zealand

## Abstract

This study examined spatial relationships between rocky shore polychaete assemblages and environmental variables over broad geographical scales, using a database compiled within the Census of Marine Life NaGISA (Natural Geography In Shore Areas) research program. The database consisted of abundance measures of polychaetes classified at the genus and family levels for 74 and 93 sites, respectively, from nine geographic regions. We tested the general hypothesis that the set of environmental variables emerging as potentially important drivers of variation in polychaete assemblages depend on the spatial scale considered. Through Moran's eigenvector maps we indentified three submodels reflecting spatial relationships among sampling sites at intercontinental (>10000 km), continental (1000–5000 km) and regional (20–500 km) scales. Using redundancy analysis we found that most environmental variables contributed to explain a large and significant proportion of variation of the intercontinental submodel both for genera and families (54% and 53%, respectively). A subset of these variables, organic pollution, inorganic pollution, primary productivity and nutrient contamination was also significantly related to spatial variation at the continental scale, explaining 25% and 32% of the variance at the genus and family levels, respectively. These variables should therefore be preferably considered when forecasting large-scale spatial patterns of polychaete assemblages in relation to ongoing or predicted changes in environmental conditions. None of the variables considered in this study were significantly related to the regional submodel.

## Introduction

Explaining the causes of variation in biodiversity at multiple spatial scales is a major goal of ecology. The ability to relate these fluctuations to changes in environmental drivers is becoming increasingly important to understand the consequences of human domination of the biosphere [1]–[3]. The scales of influence of environmental drivers, including natural and anthropogenic ones, range from the individual organism, as for the accumulation of contaminants, to the planetary scale as in the case of climatic variables [4]–[5].

Most ecological spatial studies span from the local scale, defined by the distribution of replicated observations within sites (usually 10s to 100s of m apart), to the regional scale defined by a collection of sites within a region (10s to 100s of km apart). There are several reasons to examine ecological spatial variation at these scales. First, there is ample evidence indicating that small-scale spatial heterogeneity is ubiquitous in natural populations and assemblages (e.g., [6]). Second, some of the processes accounting for local spatial patterns may also affect assemblages at larger scales. For example, local processes such as biotic interactions, behavior and fine-grain environmental heterogeneity may also propagate in space to generate large-scale patterns [7]–[8]. When patterns in species richness are of concern, regional processes, such as geographic, historic and evolutionary events may determine local species pools and their interactions [9]. Elucidating variation at one scale may therefore help understand variation at other scales. Third, most populations are generally managed locally or regionally, so these scales are relevant for practical purposes of species conservation (e.g. [10]). Finally, the analysis of spatial patterns makes sense only within the geographic limits of focal taxa distribution, setting a natural upper bound to the breadth of ecological spatial analyses.

Of course there are exceptions to the general trend of ecological spatial studies being conducted at the local and regional scales. For example, investigations examining latitudinal gradients in species richness and the distribution of migratory species and large predatory fish are often conducted at the continental or global scales [11]. These studies are becoming increasingly important to understand the consequences of biotic homogenization, where rapid changes in climatic conditions, human alteration of natural habitats and species introductions make natural barriers to organism's distribution more permeable [12]–[14]. Relating environmental and biological data over very broad spatial scales may therefore help forecast the ecological consequences of these changes. For example, knowledge of how species and assemblages distribute along temperature gradients is key to forecast the consequences of global warming on species distributional ranges and interactions [15]–[16]. While the relationship between temperature and macroecological patterns is well established for many taxa, a similar understanding has remained elusive for other environmental variables.

We examined the spatial relationships between rocky shore polychaete assemblages and environmental variables over broad geographical scales, using the database compiled by the Census of Marine Life NaGISA (Natural Geography of In Shore Areas) research program. We tested the general hypothesis that the set of environmental predictors emerging as potentially important drivers of variation in assemblages depended on the spatial scale considered. This hypothesis reflected the view that the processes maintaining differences in assemblages over large geographical scales are different from those accounting for variability at smaller scales (e.g. [17]). The opposite scenario is the one in which the same set of environmental predictors can explain variation in assemblages over a broad range of scales (e.g. [18]). In either case our study aimed at identifying an appropriate subset of variables to forecast patterns in polychaete assemblages in relation to ongoing or predicted changes in environmental conditions.

## Materials and Methods

### Biological data

**Figure 1:**
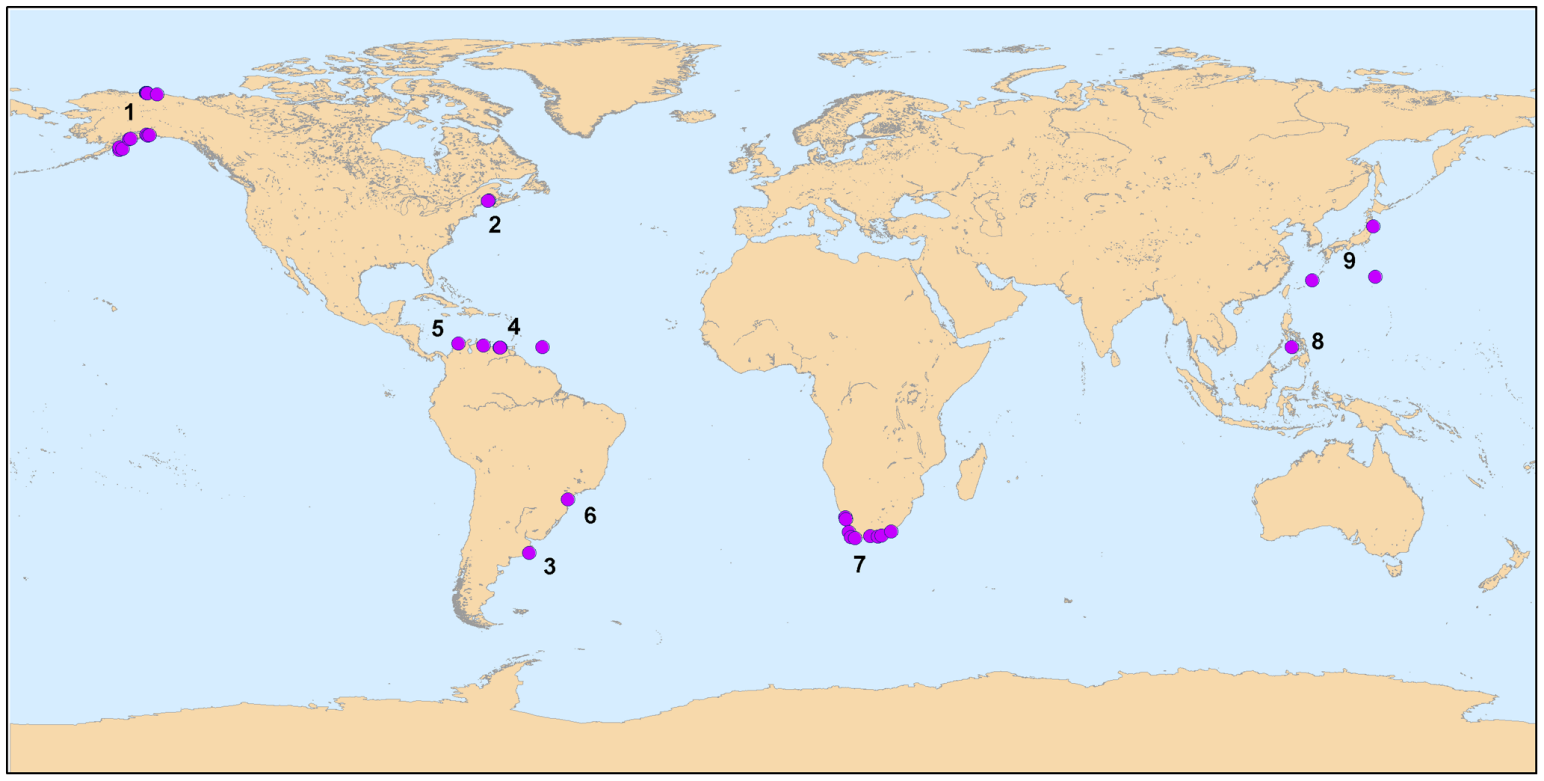
Distribution of sampling sites of polychaete assemblages. Numbers identify geographic regions (1, Alaska; 2, Canada-Maine; 3, Argentina; 4, Venezuela; 5, Colombia; 6, Brazil; 7, South Africa; 8, Philippines; 9, Japan). Several of the locations of individual sites within a region are superimposed on each other and cannot be distinguished at this scale (e.g. sites at the border between Canada and Maine). See supplementary Table S1 for further details.

Polychaetes were sampled between 2003 and 2008 in algal-dominated intertidal and shallow subtidal rocky shore habitats at 188 globally distributed sites, according to the standardized protocol developed by NaGISA [19]. The original sampling design consisted of five replicate quadrats of 25×25 cm scraped clean of all organisms at high, mid and low intertidal heights and at 1, 5, and 10 m depths at each site. Samples were washed and sieved *in situ* (mesh size of 0.5 mm) and preserved in 5% buffered formalin. Individual polychaetes were sorted and identified taxonomically in the laboratory. Due to logistical and taxonomic constraints, the spatial and temporal replication and the level of taxonomic resolution differed among sites, with samples being sorted at various taxonomic levels (species, genus or family). Because this paper focused mostly on large scale spatial patterns, we pooled samples across depth strata (intertidal and subtidal) and sampling years within sites and we examined abundance data at the genus and family levels. Only sites that included at least five quadrats per year were used in the analysis. With this restriction, 74 and 93 sites from nine geographical regions were retained for the analysis at the genus and family level, respectively (Fig. 1, Supplementary Table S1). The restricted data set included 211 genera and 55 families.

### Environmental data

For each site we collected estimates of the long-term mean values of three natural and nine anthropogenic environmental variables that could plausibly influence the distribution of polychaetes. Natural variables included sea surface temperature (SST), primary productivity (PP) and Chlorophyll-a density (CHA). For SST we used the climatological mean value for the summer season, averaged between 1985 and 2001, derived from the 4 km resolution AVHRR Pathfinder Project version 5.0 by the NOAA NODC [20]. Mean net PP, expressed as mg carbon per m^2^ per day, was estimated from the Vertically Generalized Production Model (VGPM) for SeaWiFS by the OSU Ocean Productivity Lab, spanning years from 1997 to 2007 with a 18 km resolution [21]. CHA data were derived from SeaWiFS reprocessing 5.2 by the NASA GSFC Ocean Color Group and were averaged between 1997 and 2009 with a 9 km resolution [22].

Anthropogenic variables included indexes of ocean acidification (AC), ultraviolet radiation (UV), shipping activity (SH), invasive species incidence (INV), human population density in coastal areas (HUM) and various sources of pollution, including inorganic (INP), organic (ORP), marine-derived (MARP) and nutrient contamination (NUTC). These variables were obtained by sampling 1 km resolution global maps of anthropogenic impacts provided by Halpern *et al.* [3] and were expressed as indexes ranging between 0 and 1. AC was estimated from the variation of aragonite saturation state of the ocean between 1870 and 2000–2009, while the UV index reflected the number of anomalously high values in 2000–2004 compared to 1996–1999, derived from the GSFC TOMS EP/TOMS satellite program by NASA. AC and UV had a resolution of one degree latitude/longitude (approximately 111 km in longitude at the equator or in latitude everywhere). The SH index estimated commercial ship traffic between 2004 and 2005, with data collected from the WMO Voluntary Observing Ships Scheme by NOAA, while INV was based on cargo traffic at ports and relied on data collected between 1999 and 2003. For HUM, LandScan 30 arc-second population data of 2005 were used, while INP reflected urban runoff estimated from land-use categories defined by the US Geologic Survey (http://edcsns17.cr.usgs.gov/glcc/) between 2000 and 2001. ORP and NUTC were obtained from the FAO national statistics (1992–2002) and were based on the average annual use of pesticides and fertilizers (http://faostat.fao.org). MARP was proportional to commercial shipping traffic and was derived from port data collected between 1999 and 2005.

The general approach to retrieve these data was to overlay global maps of sampling sites and abiotic variables and to directly extract values with the Nearest Neighbour algorithm using the Marine Geospatial Ecology Tools in ArcGIS (http://code.env.duke.edu/projects/mget). When satellite remote sensing data were missing for a particular site, we extracted the closest pixel value without extrapolating.

### Statistical analyses

#### Moran's Eigenvector Maps

We used Moran's Eigenvector Maps (MEM) to examine spatial variation in multivariate genus and family data and to identify the environmental variables that explained spatial pattern at multiple scales [23]. MEM is an extension of the approach known as Principal Coordinates of Neighbour matrices (PCNM) [24]. PCNM is based on the eigenvalue decomposition of a truncated matrix of geographic distances among sampling sites that is obtained through principal coordinate analysis. The truncation point usually corresponds to the smallest distance required to keep all sites connected. This procedure decomposes the spatial relationships among sampling sites into components, the eigenvectors or principal coordinate axes, which reflect variation at specific spatial scales. In general only the axes associated with positive eigenvalues are considered, with the first axes reflecting large-scale spatial structures and subsequent axes depicting variation at increasingly finer scales. However, not all axes associated with positive eigenvalues are informative and a procedure is needed to select those that contain significant spatial autocorrelation. These axes can then be used as spatial explanatory variables in univariate or multivariate regression models with biological data.

The eigenvalues resulting from the decomposition of the truncated matrix of geographic distances are linearly related to Moran's *I* coefficients of spatial autocorrelation [23]. Hence, Moran's *I* statistic is used to identify the principal coordinate axes that reflect significant spatial autocorrelation. In this context PCNM is a special case of MEM. PCNM can be extended to the more general framework defined by MEM in two important ways. First, different neighbour networks can be used to define the connectivity matrix among sampling sites, rather than using the truncation distance. Second, one can define different spatial weighting functions to weight the connections among sampling sites as a function of distance. Together, a connectivity matrix and a weighting function define a spatial weighting matrix that can be used as a predictor to model spatial variation of biological data. This matrix is a model of the spatial relationships among sampling sites. The possibility to define different spatial weighting functions enables great flexibility to model spatial variation of ecological data that is at the core of MEM.

Choice of a spatial weighting matrix is a critical step that affects the outcome of the analysis. Dray *et al.* [23] have suggested a data driven approach to select a weighting matrix that is useful in the absence of a clear theory to define and weight spatial connections among sites (e.g. dispersal or propagation processes). The approach consists of the following steps: (1) define different combinations of connectivity matrices and weighting functions, (2) compute MEM for each of these models, (3) use a multivariate analogue of multiple regression like redundancy analysis (RDA, [25]) to regress each model on multivariate biological data and retain the set of eigenvectors that result in the most appropriate model according to the corrected Akaike Information Criterion (AICc, [26]) and (4) select the model with the lowest AICc.

Once an appropriate spatial weighting matrix is identified, the eigenvectors associated with the corresponding MEM can be grouped into submodels on the basis of the similarity of their range. The range is computed by fitting a variogram model and reflects the scale at which each eigenvector depicts spatial variation. Different submodels can therefore be constructed to reflect spatial variation at different scales. Each submodel then becomes a response matrix in a multivariate regression approach (e.g. RDA) with environmental variables as predictors.

We examined four ways of defining neighbour networks [23], [27]: Delaunay triangulation, Gabriel graph, relative neighbourhood graph and distance criterion. In the last case two sites *i* and *j* were considered as neighbours if *d_ij_*<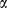, where *d_ij_* is Euclidean distance between sites and *α* is the threshold distance [23]. Inspection of the variogram suggested a value of *d_ij_* around 50; we then considered ten values of 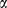 equally spaced between 40 and 60 in the analysis.

We assumed that similarity in assemblages decreased with distance according to the function 
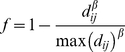
, were 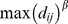 is the maximum distance defined within a given neighbour network and 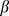 is a parameter. We examined integer values of 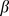 equally spaced between 1 (indicating a linear decay of similarity with distance) and 10 (allowing for different concave-down spatial relationships; preliminary analyses indicated that concave-up functions were not appropriate). We computed MEM for all combinations of binary connectivity networks and spatial weights and identified the most appropriate spatial weighting matrix according to AICc. An exponential variogram was fitted to each of the eigenvectors computed from the selected weighting matrix to estimate their range. Eigenvectors were then grouped into three submodels, reflecting spatial variation at scales >10000, between 1000 and 5000 and between 20 and 500 km, respectively.

#### Relation between environmental variables and spatial variation

To identify which environmental drivers were significantly related to variation in polychaete assemblages, we first regressed the multivariate genus and family data over environmental variables in a non-spatial RDA (i.e. without distinguishing among spatial scales). We then examined scale-specific relationships by regressing the three spatial submodels over environmental variables in separate RDAs [25]. The variance inflation factor was used to assess linear dependencies among the original covariates and only those with a variance inflation factor less than five were retained for subsequent analyses. Biological data were Hellinger-transformed before the analysis and the effects of sampling effort (number of years sampled and total number of replicates) were assessed first. After partialling out the differences in sampling effort among sites, the biological data were detrended through RDA on *X* and *Y* geographic coordinates to remove the effect of a linear spatial gradient. Analyses were done using libraries spacemakeR, vegan and spdep in R2.10 [28].

Additional analyses were performed to assess the robustness of results to three potential biases: (1) differences in sampling intensity among sites, (2) spatial and temporal confounding effects and (3) inaccuracy of satellite-derived data to characterize the nearshore environment. To account for differences in sampling intensity among sites, we used the number of pooled quadrats and sampled years within sites as covariates in all analyses. To account for spatial and temporal confounding effects, we repeated the analysis at the genus and family levels by performing a RDA with year as a covariate, using only those sites that were sampled in multiple years both in the intertidal and the subtidal and excluding regions that were sampled only at one site. The residuals obtained from these analyses were averaged within sites and used as response variables in the spatial analysis with environmental covariates. Finally, to assess whether results were robust to biases inherent in satellite-born data, we repeated the analysis based on residuals by including as predictors only those environmental variables that reflect specific human pressures in the coastal environment. We also included SST as a covariate in these analyses, since this variable poses no particular problem of estimation along shorelines [20].

## Results

Differences among sites in sampling effort (number of sampled years and replicated samples within years) were significant and accounted for 9% and 7% of variation in genus and family data, respectively (RDA). There were also significant linear spatial trends, accounting for 13% and 16% of variation at each of the two levels of taxonomic resolution, respectively.

The spatial weighting matrix with the lowest AICc value was the one originating from the distance criterion in analyses of both genera and families, with a maximum Euclidean distance to define neighbours of 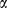 = 41 (Table 1). The selected weighting function was the one reflecting a concave-down (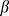 = 3) and a linear (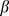 = 1) decay of similarity with distance for genera and families, respectively. Fifteen and 24 Moran's eigenvectors were retained as descriptors of spatial pattern for the two levels of taxonomic resolution, accounting for 56% and 62% of variation in the biological data, respectively (Table 1).

**Table 1:** Performance of different neighbour networks for the specification of the spatial weighting matrix.

		Genus				Family			
Connectivity	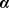	AICc	Nvar	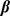	%EV	AICc	Nvar	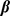	%EV
Delaunay	291	−39.2	6	1	28.1	−60.2	8	2	25.8
Gabriel	104	−36.7	6	10	25.6	−58.4	6	6	20.3
Relative	104	−38.0	7	2	29.3	−62.1	10	1	31.1
Nearest distance	41	−50.2	15	3	56.3	−72.2	24	1	61.6

%EV: percentage of explained variance; Nvar: number of variables; 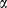 is the threshold Euclidean distance below which two sites are considered as neighbours; 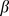 is the parameter of the spatial weighting function influencing how similarity decays with distance.

**Figure 2:**
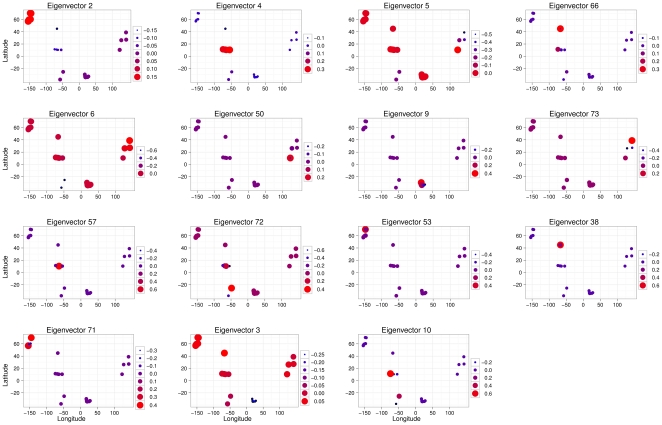
Eigenvector maps. Geographical representation of the eigenvectors used to define the spatial submodels for polychaete genera at the intercontinental (eigenvectors 2, 4, 5, 6 and 3), continental (eigenvectors 9, 73, 72, 71 and 10) and regional (eigenvectors 66, 50, 57, 53 and 38) scales. Eigenvectors are plotted in decreasing order of importance (amount of explained variance) from left to right and from top to bottom.

Three submodels, reflecting variation at different spatial scales, originated from each of the two spatial weighting matrices selected by the AICc criterion. These submodels were identified by computing the range of each eigenvector through an exponential variogram and grouping the eigenvectors with a similar range (Supplementary Figures S1 and S2). We identified an intercontinental scale (>10000 km), a continental scale (between 1000 and 5000 km) and a regional scale (between 20 and 500 km). We note that these scales are larger than the spatial resolution at which most environmental variables were obtained (between 1 and 18 km); exceptions included ocean acidification and UV radiation, which were obtained at a resolution of one degree. Eigenvectors are mapped for genera (Fig. 2) and families (Supplementary Fig. S3) to illustrate the different scales perceived.

**Figure 3:**
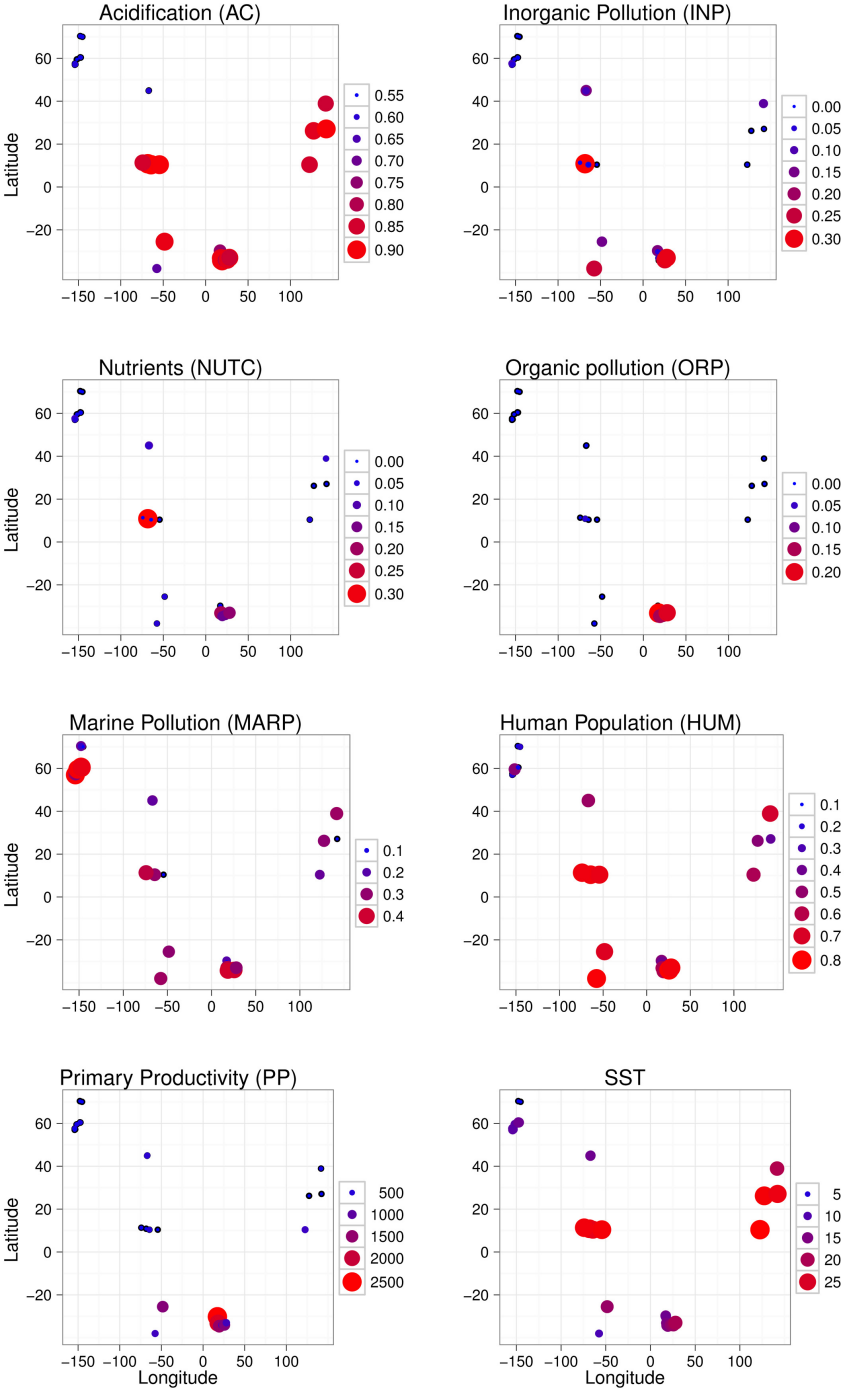
Maps of environmental variables used in the analysis.

**Figure 4:**
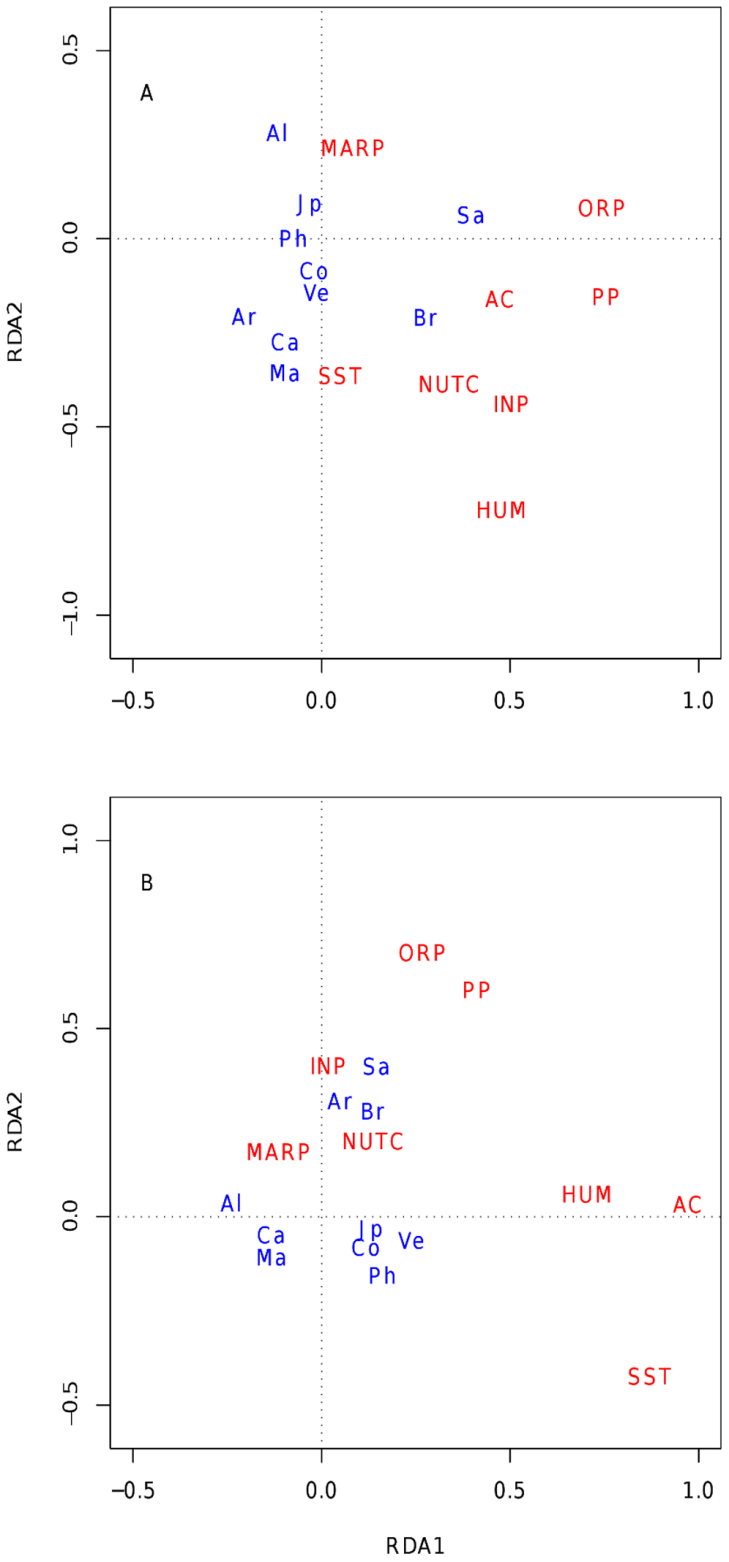
RDA plots. These illustrate the association between region centroids and environmental variables for (a) genera and (b) families. Regions include: Alaska (Al), Canada (Ca), Maine (Ma), Argentina (Ar), Venezuela (Ve); Colombia (Co), Brazil (Br), South Africa (Sa), Philippines (Ph), Japan (Jp). Environmental variables include: Acidification (AC), inorganic pollution (run-off, INP); nutrient contamination (fertilizers, NUTC); organic pollution (pesticides, ORP); marine pollution (proportional to commercial shipping traffic, MARP); human population (HUM); sea-surface temperature SST).

Eight of the 12 original environmental variables were retained after accounting for linear dependency through the variance inflation factor (Fig. 3). These variables accounted for 22% and 17% of variation in a non-spatial analysis of genus and family data, respectively (Table 2). With the exception of inorganic pollution (INP) and marine-derived pollution (MARP) all other environmental variables contributed significantly to spatial variation in polychaete genera (Table 2). All variables with the exception of INP contributed significantly to the intercontinental spatial submodel for genus data, accounting for 54% of the variation (Table 2). A plot of the first two RDA axes for this submodel illustrated the relationships among environmental variables and the centroids of sites for each of the nine regions (Fig. 4a). A positive correlation among ocean acidification (AC), organic pollution (ORP) and primary productivity (PP) and between these variables and the centroids of sites for Brazil and South Africa was evident along the first axis of the plot. Along the second axis, Canada and Maine (one region) were related to the negative scores of sea surface temperature (SST), whereas Alaska was related to marine-derived pollution as reflected by ship traffic (MARP). INP, nutrient contamination (NUTC), ORP, MARP and primary productivity (PP) were significantly related to the continental submodel, while no variable contributed significantly to the regional spatial submodel (Table 2).

**Table 2:** Pseudo-*F* values from RDA analyses relating environmental variables to polychaete data in a non-spatial regression (i.e. without distinguishing among spatial scales) and in each of the three spatial submodels originating from the spatial weighting matrices selected for genera and families.

			Spatial scale		
	Variables	Non-spatial	Intercontinental	Continental	Regional
**Genera**	AC	3.6**	16.7**	1.7	0.1
	INP	0.9	2.1	2.8*	0.6
	NUTC	1.8*	3.6*	4.4*	0.1
	ORP	3.1**	7.7**	6.3**	0.1
	MARP	1.4	2.9*	2.6*	0.1
	HUM	2.4**	7.6**	1.6	0.1
	PP	3.1**	9.9**	7.7**	0.3
	SST	1.7*	6.7**	1.8	0.1
	**%EV**	**21.8**	**53.6**	**24.8**	**1.6**
**Families**	AC	1.9**	16.0**	1.5	0.3
	INP	1.1	2.0	4.5**	0.6
	NUTC	2.3**	5.3**	10.1**	0.2
	ORP	3.0**	7.6**	8.7**	0.4
	MARP	1.2	4.0**	1.4	0.4
	HUM	2.2**	7.1**	2.7	0.2
	PP	3.4**	9.3**	11.1*	0.1
	SST	1.3	10.2**	1.6	0.2
	**%EV**	**17.1**	**53.2**	**31.6**	**2.9**

%EV: percentage of explained variance; Intercontinental scale: >10000 km; Continental scale: 1000–5000 km; Regional scale: 20–500 km. Codes for variables (and resolution in km): AC: acidification (1 degree latitude/longitude, approximately 111 km in longitude at the equator or in latitude everywhere); INP: inorganic pollution (1 km); NUTC: nutrient contamination from (fertilizers, 1 km); ORP: organic pollution (pesticides, 1km); MARP: marine pollution (proportional to commercial shipping traffic, 1km); HUM: human population data (1 km); PP: primary productivity data (18 km); SST: sea-surface temperature (4 km).

*, *P*<0.05;

**, *P*<0.01.

The analysis of family data highlighted AC, NUTC, ORP, HUM (human population) and PP as significant environmental variables (Table 2). All variables but INP were significantly related to the intercontinental spatial submodel, explaining 53% of variation (Table 2). A plot of the first two RDA axes for this submodel indicated that Alaska was positively related to NUTC and that Argentina, Brazil and South Africa were positively related to INP (Fig. 4b). No other clear pattern of association emerged from this plot. The environmental variables that were significantly related to the continental submodel were INP, NUTC, ORP and PP, while no variable contributed significantly to the regional submodel for family data, similarly to what observed for genera (Table 2).

Three regions, Argentina, Colombia and Brazil, were sampled only at one site and the first two included only subtidal data, whereas Philippines and Brazil were sampled only in one year (Table S1). To assess the extent to which our results were robust to spatial and temporal confounding effects, we performed a new analysis excluding these regions and controlling for year effects (see Methods: *Relation between environmental variables and spatial variation*). We found that temporal variation explained only 4% and 6% of variance in abundance of genera and families, respectively. Results were qualitatively similar to those obtained in the original analysis, with the strength of the relationship between environmental predictors and polychaete assemblages decreasing from the intercontinental to the regional scale (Supplementary Table S2 and S3). We note, however, that controlling for spatial and temporal confounding effects increased the percentage of explained variance compared to the original analysis. There were also some changes in patterns of significance, particularly at the continental scale, with AC and HUM becoming significant predictors for both genera and families and INP and NUTC becoming not significant in the analysis of families (Supplementary Table S3). The qualitative nature of the results remained unchanged when only environmental variables reflecting human pressures in the nearshore environment were included as predictors in the analysis (Supplementary Table S4).

## Discussion

We related spatial variation in polychaete assemblages at the genus and family levels to several potentially important environmental drivers. All drivers analyzed with the exception of inorganic pollution (INP) explained a large and significant proportion of variation of the intercontinental submodel for both genera and families (54% and 53%, respectively). INP, nutrient contamination (NUTC), organic pollution (ORP), marine-derived pollution (MARP) and primary productivity (PP) were significantly related to spatial variation of genera at the continental scale, explaining 25% of the variation. The same variables with the exception of MARP were significantly related to spatial variation of families, accounting for 32% of variability. This subset of environmental drivers had therefore the potential to explain spatial variation in polychaete assemblages at scales ranging from 1000 to >10000 km. Our results indicate that there was no clear distinction between environmental variables accounting for ecological variation at continental and intercontinental scales for genera. For families, in contrast, the environmental predictors accounting for spatial patterns at the continental scale were a subset of those explaining intercontinental variation after controlling for spatial and temporal confounding (Supplementary Table S3), None of the variables considered in this study were significantly related to the regional submodel.

Few investigations have related spatial variation in benthic assemblages to environmental explanatory variables at multiple scales. An example is provided by the study of Hewitt and Thrush [29] on the spatial and temporal distribution of macrofauna in an estuaries system in New Zealand. These authors distinguished between fine-scale and coarse-scale environmental variables and compared the relative importance of these variables in describing spatial and temporal variation of species abundance using generalized linear models. Results indicated that, in general, models combining fine-scale and coarse-scale environmental variables explained a larger proportion of variation in macrofauna assemblages than models based on one or the other type of variable alone. Broitman and Kinlan [30] examined the scales of spatial association among kelp biomass, chlorophyll *a*, SST and coastal topography along rocky shores between Baja California and Oregon. Using variograms, they found remarkably similar spatial patterns between kelps and chlorophyll *a* and this relationship was apparently driven by topographic forcing of coastal upwelling. Broad-scale intercontinental spatial variation in the structure of rocky shore upwelling ecosystems was examined by Blanchette *et al.* [31]. These authors compared the diversity and trophic structure of intertidal assemblages over a large number of sites in four geographic regions influenced by upwelling. They found an inverse relationship between environmental variability (measured as the fraction of variance in SST contained in the seasonal cycle) and the number of species across trophic levels, suggesting that species diversity is relatively low in predictable, strongly seasonal environments.

In our study, ocean acidification (AC), NUTC, ORP, human population (HUM) and PP were significantly related to spatial variation in both genera and families at one or both the intercontinental and continental scales. Several studies have documented changes in composition and abundance of macrofaunal assemblages in eutrophic conditions at local spatial scales [32]–[33]. High levels of PP and NUTC generally imply increased food availability for different trophic groups. Similarly, there is large evidence that organic pollution affects macrofauna assemblages in general [34] and polychaetes in particular [35] at small spatial scales. Our study shows that these relationships hold when examined over continental or intercontinental scales, suggesting that nutrient contamination and pollution can affect macrofauna assemblages over much larger areas than currently thought. The significant relationship with HUM further stresses the general association between polychaete assemblages and environmental conditions at broad spatial scales.

Much less is known about the relationship between spatial variation in benthic assemblages and acidification. Correlative analyses suggest that decreasing pH may impact calcareous species directly, while inducing long-term changes in abundance and of non-calcareous species through indirect effects [36]. Additional correlative evidence comes from a study examining spatial relationships between estuarine macrofauna assemblages and acid sulphate run-off associated with the Richmond River in NSW, Australia [37]. This study highlighted a negative correlation between the abundance of some polychaete species and pH in the estuary, although this pattern was probably mediated by variation in soluble aluminium concentration. Hence, despite increasing concern about the ecological consequences of ocean acidification [38] and accumulating evidence indicating that temporal fluctuations in pH affect the dynamics of marine organisms [39], little is known about how large-scale spatial variation in acidification relates to changes in marine assemblages.

Direct causal evidence of ecological effects of acidification on macrofauna assemblages comes from a mesocosm experiment where exposure to acidified conditions reduced diversity and altered species composition compared to controls [40]. These effects likely reflected variation in the physiological ability of different organism to buffer extracellular pH. However, no individual taxon emerged as particularly sensitive or particularly tolerant to reduced pH to be considered as ‘indicator’ of acidified conditions. Similarly, the association between AC and polychaete assemblages documented in our study reflected changes in the relative abundance of widespread genera and families, rather than in the presence-absence of ‘indicator’ taxa. The dominant genera in Brazil and South Africa, the regions associated with the AC index in the RDA plot (Fig. 4a), included *Lumbrineris*, *Magelona, Gunnarea*, *Pomatoleios* and *Dodecaceria*. Postulating a mechanism whereby acidification should have favored these geographically distributed genera remains problematic at this stage. It should be noted that AC was also related to SST in the RDA plot for families (Fig. 4b), reflecting a known correlation between these variables. However, we assessed the influence of each predictor variable after accounting for the effects of other covariables, such that AC was significant after controlling for variation in SST.

Our analyses highlighted similar patterns of association between environmental variables and polychaete assemblages at the genus and family levels, indicating that the coarser level of taxonomic resolution can be used to describe spatial variation at the finer level. The use of broad taxonomic categorizations as surrogates to infer spatial or temporal pattern of variability for species or genera is desirable to reduce sorting time, to increase taxonomic accuracy and to improve the efficiency of any sampling design. This is known as taxonomic sufficiency, a problem that has received a great deal of attention in the context of biodiversity assessment and in the analysis of environmental impacts [41]–[43]. Several studies have shown that high level taxa can indeed be used as surrogates for species or genera in spatial analyses at the local or regional scale (e.g. [44]–[45]; but see [46] for a different example). Our results suggest that the concept of taxonomic sufficiency may also work at very broad spatial scales.

The result indicating negligible variation at the regional scale should be taken with caution. First, environmental variables like AC and PP had a coarse spatial resolution and could never explain variation below 100 and 20 km, respectively. Second, spacing among sites did not enable detection of spatial structure below 20 km (the smallest range identified by variograms). Third, although we obtained data from nine widely distributed regions, some regions were sampled more intensively than others and samples were pooled across depth strata within sites, further reducing spatial resolution. Finally, many investigations have shown that spatial variation in marine benthic assemblages can be very large at scales ranging from metres to few kilometers (reviewed in [6]). The limited ability of our analyses to detect small-scale spatial variation could explain why environmental drivers accounted for only 22% and 17% of variation in the non-spatial analyses of genera and families, respectively.

Additional caveats must be considered when interpreting the results of investigations conducted at very large spatial scales, such as the present one. These studies often combine data collected at multiple sites over different time spans, so the potential for spatial and temporal confounding effects is large. This is particularly true when there are few spatial replicates [47]. Moreover, using satellite-derived data to characterize the nearshore environment may be problematic, particularly for those environmental variables that are estimated from the optical properties of surface sea-water [22]. We showed that when the most critical environmental variables were excluded from the analysis and when temporal variation in the most intensively sampled regions were controlled for, the qualitative nature of the results did not differ. Thus, our analyses appeared robust to likely sources of spatial and temporal confounding effects and inaccuracy of estimated environmental data.

The PCNM technique has been used to describe spatial variation in a wide range of systems, from microbial communities to forests [24], [48]–[51]. Moran's eigenvectors maps have been proposed as a generalization of PCNM [23]. We have shown that this technique was appropriate in detecting intercontinental and continental scales of variation in polychaete assemblages and in identifying the environmental variables that related to the biological data at the different scales. As we have noted, however, our sampling design was not adequate to characterize spatial patterns at small scales. While maintaining a properly replicated and balanced sampling design may be difficult when dealing with broad geographical analyses, future studies should increase replication at the site scale to allow for a more meaningful comparison between small-scale and large-scale spatial patterns.

## Supporting Information

Table S1 Polychaete sampling regions.
(0.04 MB DOC)

Table S2 Performance of different neighbour networks for the specification of the spatial weighting matrix after controlling for year effects and excluding data from regions that had only one site (Argentina, Colombia and Brazil) or that were sampled at a single point in time (Brazil and Philippines).
(0.02 MB DOC)

Table S3 Pseudo-F values from RDA analyses relating environmental variables to polychaete data in a non-spatial regression (i.e. without distinguishing among spatial scales) and in each of the three spatial submodels originating from the spatial weighting matrices selected for genera and families after controlling for year effects and excluding data from regions that had only one site (Argentina, Colombia and Brazil) or that were sampled at a single point in time (Brazil and Philippines).
(0.04 MB DOC)

Table S4 Pseudo-F values from RDA analyses relating polychaete data to environmental variables reflecting human pressure in the nearshore environment in a non-spatial regression (i.e. without distinguishing among spatial scales) and in each of the three spatial submodels originating from the spatial weighting matrices selected for genera and families after controlling for year effects and excluding data from regions that had only one site (Argentina, Colombia and Brazil) or that were sampled at a single point in time (Brazil and Philippines).
(0.04 MB DOC)

Figure S1 Maps of eigenvectors used to define spatial submodels for polychaete families at the intercontinental (eigenvectors 4, 7, 1, 10, 2, 5, 3), continental (eigenvectors 8, 12, 19, 16) and regional (eigenvectors 20, 41, 53, 51, 23, 54, 52, 92, 25, 65, 74, 50, 40) scales.
(4.78 MB TIF)

Figure S2 Exponential fits to empirical variograms of the eigenvectors used to define the spatial submodels for polychaete genera. Envelops correspond to the 0.025 and 0.975 quantiles of the distribution of 999 variograms obtained by permutation of the original data.
(1.58 MB TIF)

Figure S3 Exponential fits to empirical variograms of the eigenvectors used to define the spatial submodels for polychaete families. Envelops correspond to the 0.025 and 0.975 quantiles of the distribution of 999 variograms obtained by permutation of the original data.
(1.62 MB TIF)
