# Supplementary material for: Spatial Relationships between Polychaete Assemblages and Environmental Variables over Broad Geographical Scales
Source: PLoS One. 2010 Sep 23;5(9):e12946. doi: 10.1371/journal.pone.0012946 (PMC2944868; doi:10.1371/journal.pone.0012946)
Supplement: Table S1 — Polychaete sampling regions. (0.04 MB DOC) [file pone.0012946.s001.doc]

| **Ocean** | **Region** | **Year** | **N. Sites** | **N. Plots** | **Habitat** |
| --- | --- | --- | --- | --- | --- |
| Pacific | Alaska | 2003-2007 | 17 | 1294 | I,S |
| Atlantic | Canada/Maine | 2007-2008 | 27 | 363 | I,S |
| Atlantic | Argentina | 2007-2008 | 1 | 34 | S |
| Atlantic | Venezuela | 2007-2008 | 7 | 92 | S |
| Atlantic | Colombia | 2006-2007 | 1 | 10 | S |
| Atlantic | Brazil | 2007 | 1 | 7 | I,S |
| Atlantic-Indian | South Africa | 2007-2009 | 12 | 109 | I,S |
| Pacific | Philippines | 2007 | 22 | 209 | I,S |
| Pacific | Japan | 2006-2008 | 5 | 127 | I,S |

I: intertidal; S: subtidal
