# Supplementary material for: Spatial Relationships between Polychaete Assemblages and Environmental Variables over Broad Geographical Scales
Source: PLoS One. 2010 Sep 23;5(9):e12946. doi: 10.1371/journal.pone.0012946 (PMC2944868; doi:10.1371/journal.pone.0012946)
Supplement: Table S2 — Performance of different neighbour networks for the specification of the spatial weighting matrix after controlling for year effects and excluding data from regions that had only one site (Argentina, Colombia and Brazil) or that were sampled at a single point in time (Brazil and Philippines). (0.02 MB DOC) [file pone.0012946.s002.doc]

|  |  | **Genus** | | | | **Family** | | | |
| --- | --- | --- | --- | --- | --- | --- | --- | --- | --- |
| **Connectivity** | ***α*** | **AICc** | **Nvar** | ***β*** | **%EV** | **AICc** | **Nvar** | ***β*** | **%EV** |
| Delaunay | 291 | -267.3 | 6 | 1 | 27.4 | -343.2 | 5 | 10 | 17.9 |
| Gabriel | 116 | -269.5 | 7 | 1 | 33.6 | -345.0 | 6 | 1 | 22.9 |
| Relative | 116 | -270.6 | 7 | 1 | 34.9 | -345.2 | 6 | 1 | 23.3 |
| Nearest distance | 37 | -298.2 | 19 | 1 | 82.8 | -372.0 | 21 | 1 | 75.4 |

%EV: percentage of explained variance; Nvar: number of variables; $\alpha$is the threshold Euclidean distance below which two sites are considered as neighbours; $\beta$ is the parameter of the spatial weighting function influencing how similarity decays with distance
