# Supplementary material for: Spatial Relationships between Polychaete Assemblages and Environmental Variables over Broad Geographical Scales
Source: PLoS One. 2010 Sep 23;5(9):e12946. doi: 10.1371/journal.pone.0012946 (PMC2944868; doi:10.1371/journal.pone.0012946)
Supplement: Table S3 — Pseudo-F values from RDA analyses relating environmental variables to polychaete data in a non-spatial regression (i.e. without distinguishing among spatial scales) and in each of the three spatial submodels originating from the spatial weighting matrices selected for genera and families after controlling for year effects and excluding data from regions that had only one site (Argentina, Colombia and Brazil) or that were sampled at a single point in time (Brazil and Philippines). (0.04 MB DOC) [file pone.0012946.s003.doc]

|  |  |  | **Spatial scale** | | |
| --- | --- | --- | --- | --- | --- |
|  | **Variables** | **Non-spatial** | **Intercontinental** | **Continental** | **Regional** |
| **Genera** | AC | 3.3** | 13.7** | 6.5** | 0.3 |
|  | INP | 1.8 | 1.5 | 35.5** | 1.2 |
|  | NUTC | 2.1* | 4.1* | 25.7** | 1.1 |
|  | ORP | 1.8 | 8.1** | 14.2** | 1.4 |
|  | MARP | 1.6 | 4.5* | 8.5** | 0.4 |
|  | HUM | 1.5 | 9.4** | 11.1** | 0.2 |
|  | PP | 2.8** | 5.4* | 37.0** | 1.8 |
|  | SST | 1.4 | 8.3** | 9.9** | 0.2 |
|  | **%EV** | **24.1** | **60.0** | **66.9** | **12.6** |
| **Families** | AC | 1.7 | 7.9** | 8.9** | 0.6 |
|  | INP | 1.8 | 27.0** | 1.5 | 1.7 |
|  | NUTC | 2.8* | 31.0** | 1.4 | 0.9 |
|  | ORP | 2.4* | 38.9** | 4.6* | 0.5 |
|  | MARP | 1.3 | 0.7 | 2.9 | 0.8 |
|  | HUM | 0.9 | 3.5* | 4.5* | 0.7 |
|  | PP | 2.8* | 68.7** | 3.8* | 0.2 |
|  | SST | 1.0 | 0.7 | 1.5 | 0.7 |
|  | **%EV** | **18.6** | **78.4** | **40.9** | **5.7** |

%EV: percentage of explained variance. Intercontinental scale: >10000 km; Continental scale: 1000-5000 km; Regional scale: 20-500 km. Codes for variables: AC: acidification; INP: inorganic pollution; NUTC: nutrient contamination (fertilizers); ORP: organic pollution (pesticides); MARP: marine pollution (proportional to commercial shipping traffic); HUM: human population data; PP: primary productivity data; SST: sea-surface temperature. *, *P*<0.05; **, *P*<0.01
