# Supplementary figures and images for: Spatial Relationships between Polychaete Assemblages and Environmental Variables over Broad Geographical Scales
Source: PLoS One. 2010 Sep 23;5(9):e12946. doi: 10.1371/journal.pone.0012946 (PMC2944868; doi:10.1371/journal.pone.0012946)

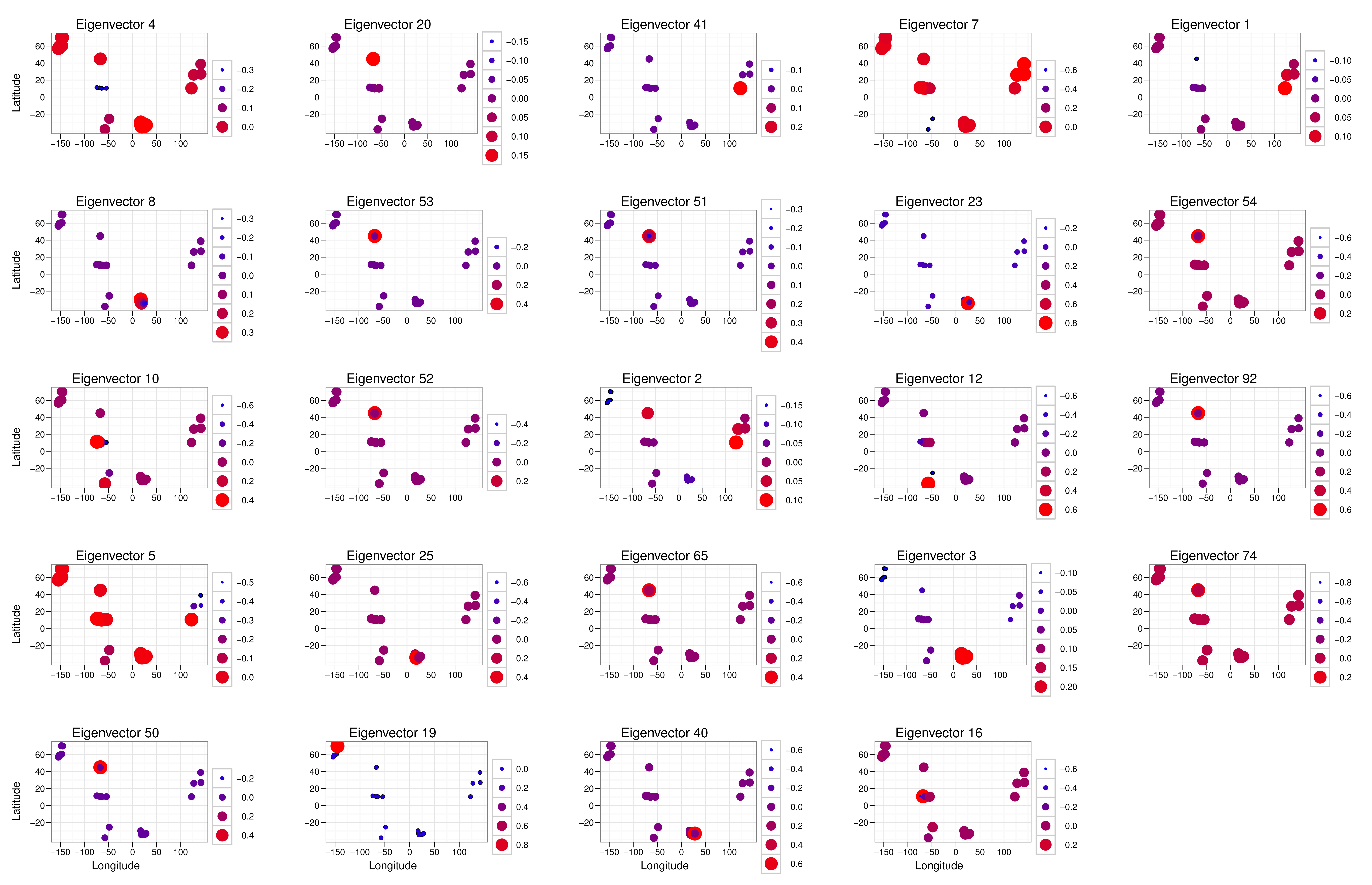

Supplement: Figure S1 — Maps of eigenvectors used to define spatial submodels for polychaete families at the intercontinental (eigenvectors 4, 7, 1, 10, 2, 5, 3), continental (eigenvectors 8, 12, 19, 16) and regional (eigenvectors 20, 41, 53, 51, 23, 54, 52, 92, 25, 65, 74, 50, 40) scales. (4.78 MB TIF) [file pone.0012946.s005.tif]

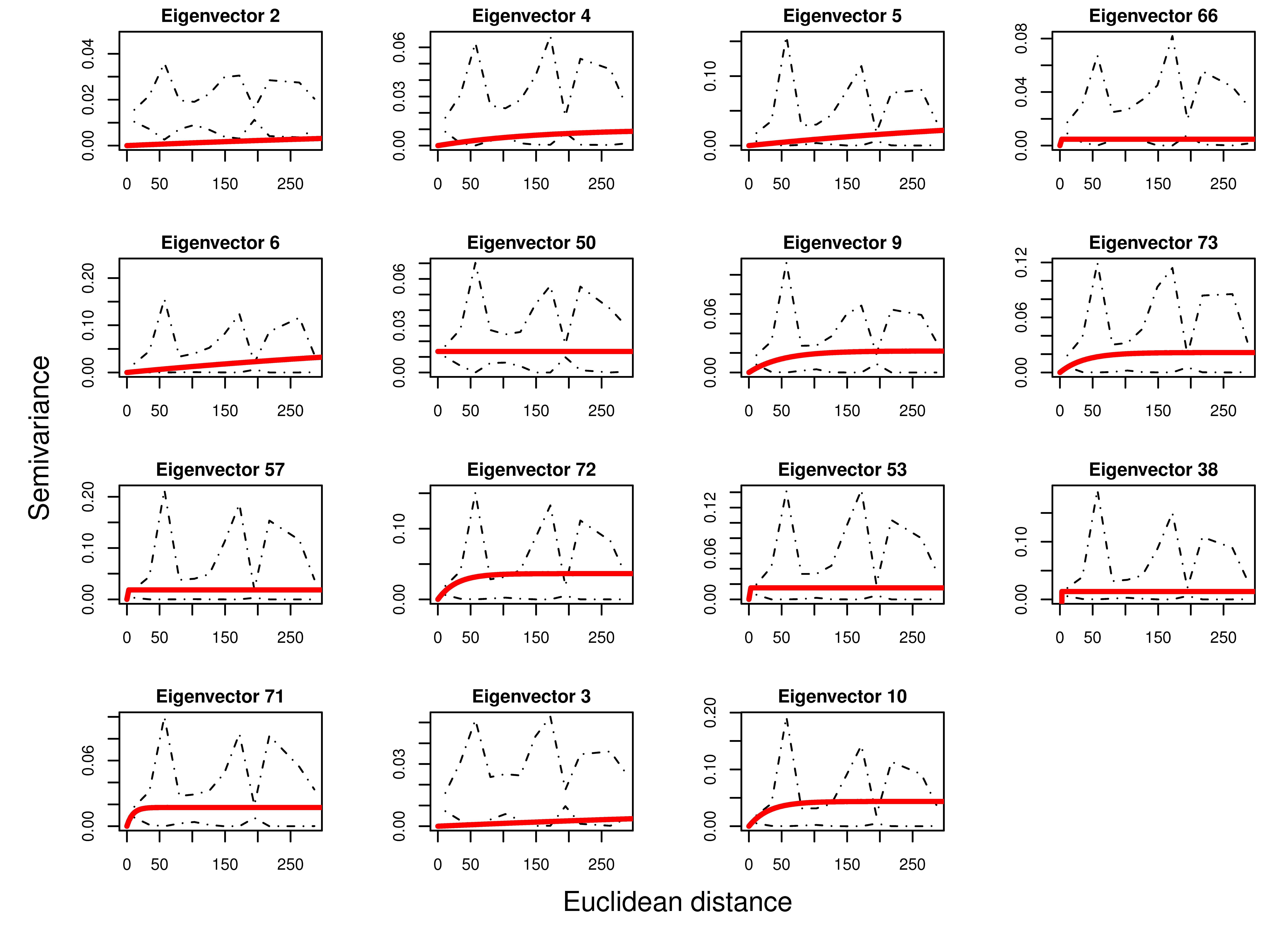

Supplement: Figure S2 — Exponential fits to empirical variograms of the eigenvectors used to define the spatial submodels for polychaete genera. Envelops correspond to the 0.025 and 0.975 quantiles of the distribution of 999 variograms obtained by permutation of the original data. (1.58 MB TIF) [file pone.0012946.s006.tif]
